# The role of depression and physical activity in the association of between sleep quality, and duration with and health-related quality of life among the elderly: a UK Biobank cross-sectional study

**DOI:** 10.1186/s12877-022-03047-x

**Published:** 2022-04-19

**Authors:** Wei Hu, Jiadong Chu, Xuanli Chen, Siyuan Liu, Na Sun, Qiang Han, Tongxing Li, Zhaolong Feng, Qida He, Yueping Shen

**Affiliations:** Department of Epidemiology and Biostatistics, School of Public Health, Medical College of Soochow University, 199 Renai Road, Suzhou, 215123 People’s Republic of China

**Keywords:** Sleep quality and duration, Health-related quality of life, Depression, Physical activity, Elderly

## Abstract

**Background:**

Although studies have shown that sleep quality (duration) is associated with health-related quality of life (HRQoL), most of these studies have been small-sized and targeted at young and middle-aged adults. In addition, few studies have explored the path mechanism of sleep disorders leading to impaired HRQoL.

**Objectives:**

This study aimed to determine the association between sleep quality and duration and HRQoL among the elderly in the United Kingdom, assess whether depression mediated the association, and explore the role of physical activity (PA) in the path association.

**Methods:**

Data were extracted from the baseline survey of the UK Biobank, a large prospective cohort study enrolling more than 500,000 participants, of which 52,551 older adults (aged ≥60 years) were included in the study. HRQoL was assessed using the European Quality of Life-5 Dimensions. Tobit and multivariate logistic regression models were used to determine the association between sleep quality and duration and HRQoL. The mediating and moderated mediation models were estimated using the PROCESS macro and MEDCURVE macro.

**Results:**

The Tobit model showed that the elderly with short or long sleep duration (β = − 0.062, 95% confidence interval [CI] = − 0.071 to − 0.053; β = − 0.072, 95% CI = − 0.086 to − 0.058) had worse HRQoL after adjusting potential covariates. In the logistic regression models, we found an inverted U-shaped association between sleep duration and HRQoL. Moreover, a significant positive association was observed between sleep quality and HRQoL (all *P* < 0.05). The results also revealed that depression mediated the association between sleep disorders and HRQoL (sleep quality: β = 0.008, 95% CI = 0.007–0.010; sleep duration: θ = 0.001 [mean], 95% CI = 0.001–0.002). Furthermore, PA moderated all paths among sleep quality and duration, depression, and HRQoL, and greater effects were observed in the elderly with lower PA levels.

**Conclusions:**

The findings show that poor sleep quality and duration were independently associated with worse HRQoL among the elderly in the United Kingdom. Furthermore, PA buffers the mediating effect of depression and adverse effects of sleep disorders on HRQoL. It is essential to properly increase PA and provide early intervention for depression in the elderly with sleep disorders to improve their HRQoL.

**Supplementary Information:**

The online version contains supplementary material available at 10.1186/s12877-022-03047-x.

## Introduction

Sleep is a fundamental behaviour that accounts for nearly a third of the day and is not only the physiological process of regulating and maintaining the health status of the body but also an important indicator of an individual’s health status [1, 2]. Sleep duration is decreasing in modern society, and unhealthy sleep habits are a growing trend [3]. More than one-third of the world’s population has sleep problems, which are particularly prominent among the elderly [4]. Sleep status generally changes with age [5]. Studies have shown that up to 50% of the elderly in Europe experience varying degrees of sleep disorders [5, 6]. Sleep disorders (such as insomnia, sleep apnoea, and snoring), often characterised by decreased sleep quality and abnormal sleep duration, are increasingly common health problems among the elderly [7, 8]. A large number of epidemiological and laboratory studies have confirmed that sleep disorders are not only associated with all-cause mortality, incidence of mental illness, and cardiovascular disease in the elderly but also lead to fatigue, weakness, and cognitive decline, which ultimately worsen an individual’s health-related quality of life (HRQoL) [9–13].

HRQoL has been widely used clinically to evaluate the effect of sleep disorders on health in the elderly population because it provides a multidimensional perspective and considers the patient’s emotional and physical functioning and social well-being [14–16]. Although previous studies have suggested that sleep disorders could have a disadvantageous influence on the HRQoL [14, 16–19], there are still some questions that have not been fully addressed. Firstly, most of them are small sample size [14, 20], and more attention is paid to young and middle-aged people [21, 22]. However, since sleep duration (quality) and HRQoL will decrease with age, so they may be less representative for the elderly. Additionally, the vast majority of studies only focus on the sleep duration [13, 17] and draw inconsistent conclusions [16, 20, 21]. Last but not least, few studies have explored the pathway mechanism of HRQoL decline caused by sleep disorders. Accordingly, it is imperative to confirm the association between sleep duration (quality) and HRQoL utilizing a large sample database, and further explore the pathway mechanism behind the association.

Further exploration of the path mechanism provides a new perspective on how and when independent variables affect the dependent variables. In this study, we regard depression as a mediator between sleep disorders and HRQoL for the following reasons: Firstly, a study of an elderly Asian population found that poor sleep quality is associated with depressive symptoms (OR = 5.14, 95% CI: 3.21 to 8.23) [23]. A study adopting longitudinal design found that long sleep duration significantly predicted depressive symptoms after 2 years (OR = 2.52, 95% CI: 1.27 to 4.99) [24]. Additionally, a number of studies have suggested that depressive symptoms are associated with low HRQoL in older adults [25–27]. For example, a study in Nepal found a significant negative correlation between depression and HRQoL (coefficient = − 1.23, 95% CI: − 1.72 to − 0.72) [25]. Hence, it is reasonable to regard depression as a mediator between sleep quality and duration and HRQoL. Empirical studies have confirmed that physical activity (PA) is a key factor in improving the health of the elderly [28–30]. For example, a randomised controlled trial showed that increased PA could improve depression mood in insomnia individuals (F (8, 26) = 5.16, *P* = 0.03) [29]. A meta-analysis indicated that PA could improve the physical and mental health of patients with depression [30]. In a randomized controlled trial of elderly people with insomnia, it was found that aerobic exercise such as walking, cycling or treadmill could ameliorate their quality of life [28]. Accordingly, we speculated that PA may moderate the direct and indirect relationships between sleep disorders and HRQoL through depression as a mediator.

Data-driven inference to determine the association between sleep quality and duration and HRQoL and whether depression and PA may be potential explanations for the association between sleep disorders and impaired HRQoL is crucial. Thus, the purposes of this study were two-fold: (1) to examine the association between sleep quality and duration and HRQoL based on a large-scale national representative database (UK Biobank [UKB]) among the elderly in the United Kingdom and (2) to explore whether depression mediated the association between sleep and HRQoL, and whether PA moderated the association between these three factors based on a theoretical framework model diagram (Fig. s1).

## Materials and methods

### Study population

The study population comprised 51,551 older (aged 60 years and over) participants of the UKB, a large-scale prospective cohort study with 502,490 participants recruited between 2006 and 2010 across the United Kingdom. A detailed description of the UKB database has been reported previously [31]. The UKB project was approved by the North West Multicenter Research Ethical Committee, and all participants provided informed consent at recruitment.

Before the analysis, we cleaned the data appropriately. First, 336,006 participants with missing HRQoL-related data were excluded based on self-reported diagnoses obtained via verbal interview. Second, 104,276 participants aged under 60 years were excluded based on their age at recruitment (UKB data field 21,022). A total of 564 participants with missing sleep quality data consisting of five sleep behaviour data were excluded. Additionally, we excluded 10,093 participants who lacked PA-related data (8517) and depression-related data (1576). Ultimately, 51,551 elderly participants in the UKB were included in this study (Fig. s2).

### Definition of sleep duration and quality

Sleep duration was defined according to the following question: ‘How many hours of sleep do you get in every 24 hours? (please include naps)?’ To obtain possible nonlinear association, sleep duration was divided into five categories: ≤5, 6, 7–8, 9, and ≥ 10 h, with 7–8 h as the reference [32]. Short sleepers were defined as participants who slept ≤5 h/d, and long sleepers were defined as participants who slept ≥10 h/d [33].

A new healthy sleep score, including five sleep characteristics, was used to evaluate the sleep quality of participants [34]. Early chronotype (‘morning’ or ‘morning than evening’) (UK Biobank code: 1180), adequate sleep duration (7–8 h/day) (code: 1160), never or rarely experience sleeplessness/insomnia (code: 1200), no self-reported snoring (code: 1210), and no frequent daytime sleepiness (code: 1220) represented low-risk sleep characteristics [34]. For each sleep characteristic, participants with low-risk sleep characteristics were assigned a score of 1, whereas those classified as high-risk had a score of 0. Participants were scored from 0 to 5, according to their number of the low-risk sleep characteristics, and were divided into three groups: ‘healthy’ (scores ≥4), ‘less healthy’ (scores at 2 and 3), and ‘poor’ (scores ≤1) [34].

### Definition of physical activity and depression

The total metabolic equivalent task minutes (UK Biobank code: 22040) were used to measure total PA (including walking and moderate and vigorous activity) in the past week [35]. In UK Biobank, the validated 2-item Patient Health Questionnaire (PHQ-2) was used to assess depressive symptoms at baseline [36]. The questionnaire asked respondents about the frequency of “depression mood” (UK Biobank code: 2050) and “unenthusiasm/disinterest” (UK Biobank code: 2060) in the past two weeks. The response options included “not at all” (assigned a score of 0), “several days” (1), “more than half the days” (2) and “nearly every day” (3). Therefore, the score range of the questionnaire can be from 0 to 6, where 3 scores and above indicated that depression may occur [36]. PA and depression were considered continuous variables in the current study.

### Health-related quality of life measure

HRQoL was measured using the European Quality of Life-5 Dimensions 5-levels (EQ-5D-5L) (UK Biobank codes: 120098–120,102) instrument consisting of a self-reported five-dimensional health descriptive system and self-rated overall health using the EuroQol visual analogue scale [37]. The descriptive system consists of five dimensions, including mobility (MO), self-care (SC), usual activities (UA), pain/discomfort (PD), and anxiety/depression (AD), and each dimension has five levels of response (from ‘no problems’ to ‘extreme problems’). The self-reported health states of the five dimensions can be converted into a single utility score based on the England value set [38]. For example, the health status “12,212” indicated that the respondent had no problems with MO and PD, but had slightly problems with SC, UA and AD. Then the utility score of respondents with health status of “12,212” =1− (0 + 0.050 + 0.050 + 0 + 0.078) = 0.822 (Table s1). The utility score ranges from − 0.281 (worst health) to 1 (full health), with higher scores indicating better HRQoL. Low HRQoL was defined as a utility score less than or equal to the mean minus one standard deviation (SD) [39]. Cronbach’s coefficient was 0.797 in the present study.

### Measurement of covariates

Sociodemographic variables included self-reported age, sex (female vs. male), race (recorded as white and others), educational level (college/university and others), socioeconomic status (SES, according to the Townsend deprivation index, an official measurement of relative material deprivation in small areas [40]), smoking status (current or former vs. never), alcohol consumption (current or former vs. never), and body mass index (BMI, kg/m^2^). Non-communicable disease (NCD) variables associated with sleep disorders, including hypertension, diabetes, cataract, stroke, coronary heart disease (CHD), chronic obstructive pulmonary disease, asthma, and migraine, were ascertained from self-reported physician diagnoses of 345 diseases encoded using the International Classification of Diseases-Tenth Revision.

### Statistical analyses

The baseline characteristics of the study participants were summarised as numbers with percentages and means ± SDs for categorical and continuous variables, respectively. Pearson’s chi-squared analysis for categorical variables and Wilcoxon’s rank-sum test for continuous variables were used to determine differences in baseline characteristics according to sleep quality and duration.

A Tobit regression model was used to explore the association between sleep quality and duration and HRQoL utility score (continuous) [41]. Multivariate logistic regression was used to evaluate the association between sleep quality and duration and HRQoL utility scores and dimensions, while controlling for the measured covariates. In this analysis, each EQ-5D dimension (with or without problems) and utility score (low or high) were regarded as binary dependent variables. In addition, three models were constructed in this study. Model 1 was adjusted for sociodemographic characteristics. Model 2 was further adjusted for NCD factors. Model 3 included all covariates in Model 2, plus PA and depression scores.

The Spearman correlation coefficient was used to examine the correlations among the main study variables. The PROCESS and MEDCURVE macro, according to Hayes’ recommendation [42, 43], were used when assessing the mediation and moderated mediation models for sleep quality and duration, depression, and HRQoL. All regression coefficients were tested using the bias-corrected percentile bootstrap (repeated sampling 5000 times), while controlling for measured covariates, and all variables were standardised prior to data analysis [44].

There were 74 types of models in the PROCESS macro [42]. In the present study, model 4 was selected to test the mediating effect, and model 59 was applied to test the moderated mediation effect between sleep quality and HRQoL. Simple slope analysis was performed to further examine the direction and intensity of the moderation effect when the moderated variable (PA) was divided into two levels, high and low, according to M (mean) ± 1 SD [45].

Curve fitting based on a generalised additive model was conducted to test the nonlinear association among sleep duration, depression, and HRQoL [39]. After confirming the nonlinear association, a mediation model was constructed using the MEDCURVE macro. The mediating effect of depression in this nonlinear association was called the instantaneous indirect effect (θ) [43]. To better understand θ, we estimated θ at M (mean sleep duration) – 1 SD, M, and M + 1 SD, in that θ changes with different levels of sleep duration [43]. Similar to sleep quality, the PROCESS macro (model 59) was also used to test the moderated mediation effect of the nonlinear association by specifying quadratic sleep duration as the independent variable, depression as the mediator variable, PA as the moderator variable, HRQoL as the outcome variable, and sleep duration and interaction between sleep duration and PA as additional control variables [46].

All data analyses were performed using SAS version 9.4 (SAS Institute Inc., Cary, NC, USA) and SPSS version 25.0 (SPSS Inc., Armonk, NY, USA). A *P* value < 0.05 was considered significant using two-sided tests.

## Results

### Characteristics of participants

Overall, the participants reported a mean HRQoL utility score of 0.883 ± 0.135, and 60.3% rated their sleep quality as less healthy. The percentage of female sex, educational level below college/university, prevalence of history of hypertension and CHD, and asthma were significantly higher among those with poor sleep quality than those with healthy and less healthy sleep quality (*P* < 0.05). Conversely, participants with healthy sleep quality were more likely to possess higher SES and HRQoL utility scores and lower BMI and depression scores than those with less healthy and poor sleep quality (*P* < 0.05) (Table 1). Regarding sleep duration, the average sleep duration of the elderly was 7.25 ± 1.00 h, and those with 7–8 h of sleep duration significantly performed better in all respects than with other sleep durations (Table s2). An inverted U-shaped association was observed between sleep duration and mean HRQoL utility score, with the highest HRQoL at 7–8 h of sleepers (Fig. s3A). A positive correlation was also observed for sleep quality (Fig. s3B).

**Table 1:** Characteristics of study participants according to sleep quality among the elderly in the UK

Baseline characteristics	Poor	less-healthy	healthy	*P*
	(*n* = 2238)	(*n* = 31,109)	(*n* = 18,204)	
Sociodemographic characteristics				
Age, years	63.6 ± 2.7	63.6 ± 2.8	63.6 ± 2.7	0.059
Female	1182(52.8)	15,737(50.6)	9093(50.0)	0.029
Townsend Index	− 1.8 ± 2.8	−1.9 ± 2.7	− 2.0 ± 2.7	0.003
College or university degree	842(37.6)	12,683(40.8)	7769(42.7)	< 0.001
Ethnicity, white race	2195(98.3)	30,525(98.5)	17,879(98.5)	0.580
BMI, kg/m^2^	27.1 ± 4.3	26.9 ± 4.2	26.8 ± 4.2	< 0.001
Sum MET, min	2550 ± 2551	2614 ± 2485	2584 ± 2407	0.022
Depression score	0.43 ± 0.95	0.33 ± 0.81	0.30 ± 0.75	< 0.001
Current or former smoker	1078(48.2)	14,817(47.6)	8515(46.8)	0.136
Current or former alcohol drinker	2164(96.7)	30,140(96.9)	17,670(97.1)	0.415
NCDs				
Hypertension	289(12.9)	3698(11.9)	1991(10.9)	< 0.001
Diabetes	115(5.1)	1453(4.7)	782(4.3)	0.063
Cataract	109(4.9)	1397(4.5)	805(4.4)	0.624
Stroke	55(2.5)	670(2.2)	368(2.0)	0.324
CHD	149(6.7)	1934(6.2)	1015(5.6)	0.006
COPD	51(2.3)	592(1.9)	326(1.8)	0.251
Asthma	166(7.6)	2420(8.0)	1259(7.1)	0.002
Migraine	85(3.8)	1051(3.4)	565(3.1)	0.110
Low-risk sleep characteristics				
Early chronotype	57(3.2)	13,141(48.7)	15,655(89.3)	< 0.001
Sleep 7–8 h/day	160(7.2)	19,675(63.3)	17,035(93.6)	< 0.001
Never/rarely insomnia	18(0.8)	4738(14.1)	8833(48.5)	< 0.001
No self-reported snoring	41(2.1)	15,063(52.4)	16,147(90.0)	< 0.001
No frequent daytime sleepiness	1893(84.9)	30,324(97.6)	18,168(99.8)	< 0.001
EQ-5D-5L				
Utility score	0.867 ± 0.147	0.881 ± 0.137	0.889 ± 0.129	< 0.001
Problems of Dimensions				
Mobility	832(37.2)	10,966(35.3)	6068(33.3)	< 0.001
Self-care	286(12.8)	3344(10.8)	1746(9.6)	< 0.001
Usual activities	932(41.6)	11,611(37.3)	6450(35.4)	< 0.001
Pain/discomfort	1351(60.4)	17,968(57.8)	10,235(56.2)	< 0.001
Anxiety/depression	588(26.3)	6922(22.3)	3658(20.1)	< 0.001

*EQ-5D-5L* European Quality of Life-5 Dimensions 5-levels, *CHD* coronary heart disease, *COPD* chronic obstructive pulmonary disease. Continuous variables presented as mean ± SD (standard deviation) and categorical variables presented as n (%)

### Association between sleep quality and duration and health-related quality of life

**Fig. 1:**
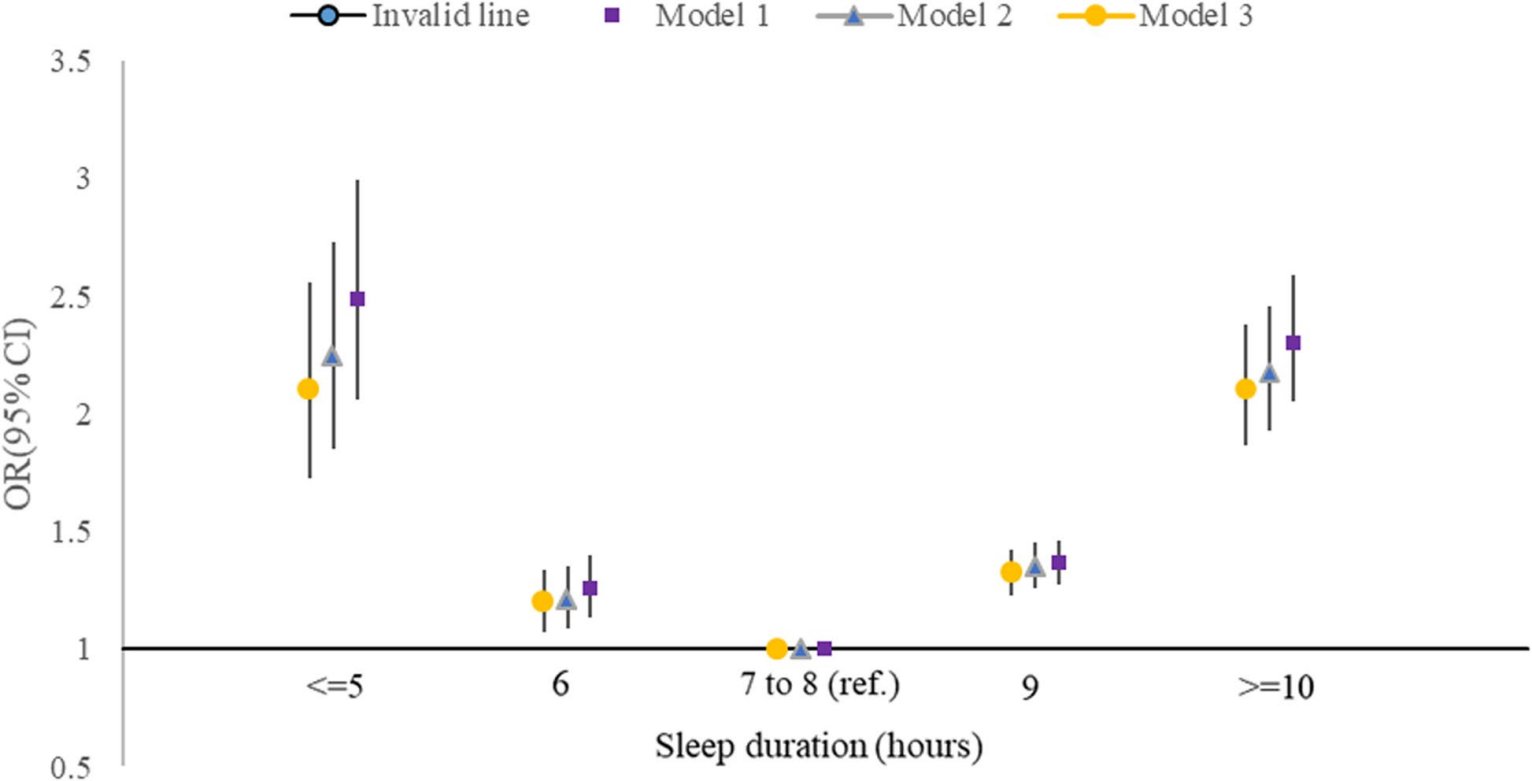
Association between sleep duration categories and low health-related quality of life among the elderly

**Fig. 2:**
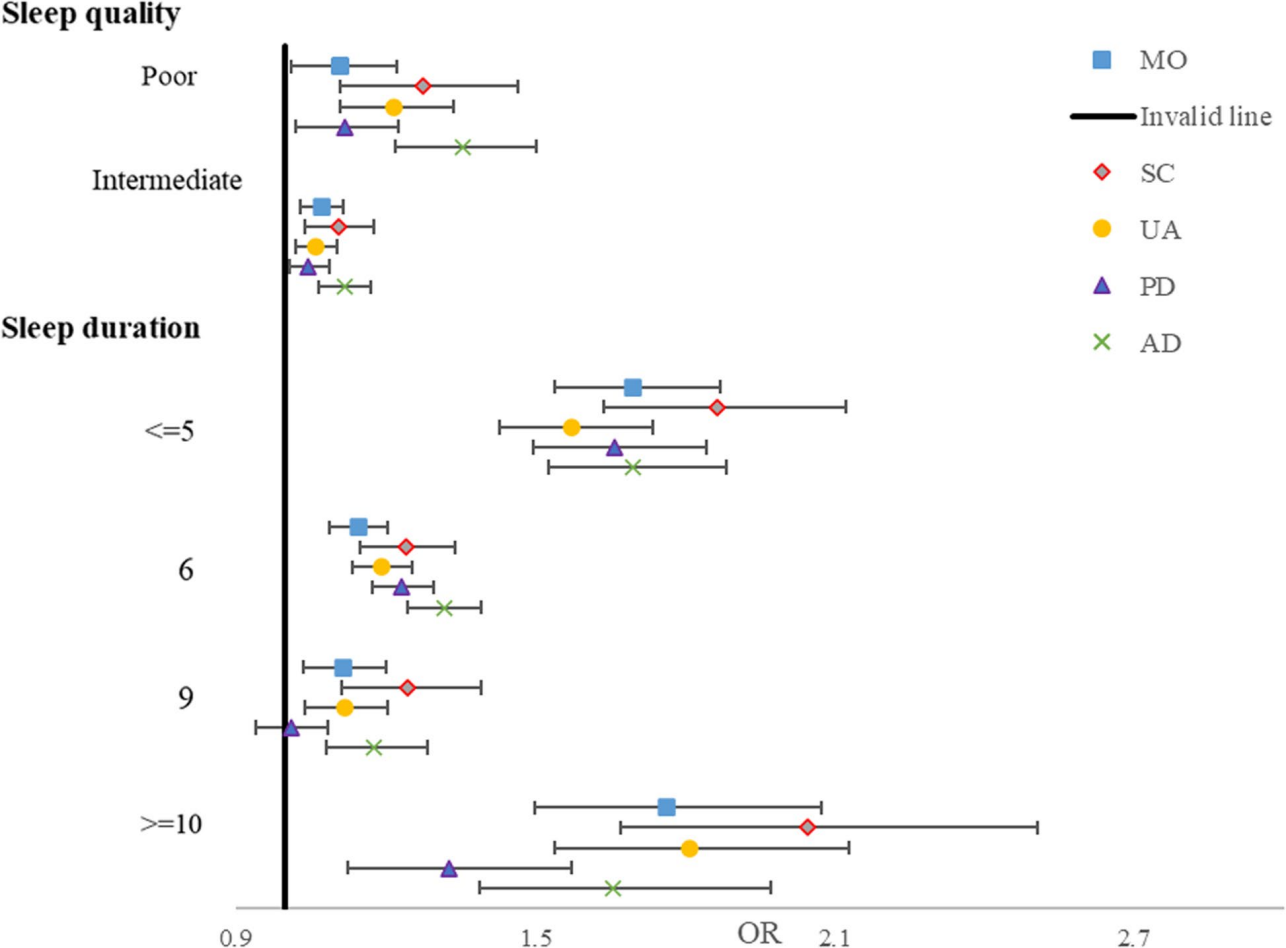
Association between sleep quality, duration, and self-reported the incidence of EQ-5D-5L problems among the elderly

HRQoL utility scores increased as sleep quality improved, although the ascent slightly weakened after more covariates were adjusted. According to model 3 (adjusted all covariates), the regression coefficients for elderly with less healthy and healthy sleep quality were 0.012 (95% CI, 0.004–0.020, *P* = 0.003) and 0.021 (95% CI, 0.012–0.029, *P* < 0.001), respectively. A curvilinear association was observed between sleep duration and HRQoL utility scores. Short sleepers had lower HRQoL (β = − 0.062; 95% CI, − 0.071 to − 0.053 for ≤5 h, *P* < 0.001), and long sleepers had lowest HRQoL (β = − 0.072; 95% CI, − 0.086 to − 0.058 for ≥10 h, *P* < 0.001) compared with 7- to 8-h sleepers (Table 2). When HRQoL was dichotomised, no more than 5 h, 6 h, 9 h, 10 h of sleep duration were all significantly inversely U-shaped and associated with increased odds for low HRQoL compared with reference group (odds ratio, OR = 2.10, 1.32, 1.20, 2.10, respectively, all *P* < 0.001), after adjustment for potential confounders (model 3) (Fig. 1). Similar results were found for sleep quality (OR = 1.11 and 1.29 for less-healthy and poor sleep quality in model 3, all *P* < 0.001) (Fig. s4). Furthermore, regarding the risk of experiencing EQ-5D problems, participants with abnormal sleep duration (OR = 1.12–2.05, except for sleep duration of 9 h on pain/discomfort dimension, all *P* < 0.001) and poor sleep quality (OR = 1.05–1.36, all *P* < 0.001) had a higher risk of problems with EQ-5D dimensions than their counterparts (Fig. 2).

**Table 2:** Association between sleep quality, duration, and the utility scores using Tobit regression analysis

	Model 1			Model 2			Model 3		
Sleep quality model	β	95%CI	*P*	β	95%CI	*P*	β	95%CI	*P*
Poor	Ref.			Ref.			Ref.		
Less-healthy	0.016	0.008, 0.024	< 0.001	0.014	0.006, 0.022	< 0.001	0.012	0.004, 0.020	0.003
Healthy	0.025	0.017, 0.033	< 0.001	0.023	0.015, 0.031	< 0.001	0.021	0.012, 0.029	< 0.001
R^2^	0.112			0.139			0.280		
Sleep duration (hours) model	β	95%CI	*P*	β	95%CI	*P*	β	95%CI	*P*
<=5	−0.070	−0.079, −0.061	< 0.001	−0.065	−0.074, −0.056	< 0.001	−0.062	−0.071, − 0.053	< 0.001
6	−0.026	− 0.030, − 0.022	< 0.001	− 0.024	− 0.029, − 0.020	< 0.001	− 0.023	− 0.027, − 0.018	< 0.001
7–8	Ref.			Ref.			Ref.		
9	−0.018	− 0.024, − 0.011	< 0.001	− 0.015	−0.021, − 0.008	< 0.001	−0.014	− 0.021, − 0.008	< 0.001
> = 10	− 0.084	−0.098, − 0.070	< 0.001	−0.077	− 0.092, − 0.063	< 0.001	−0.072	− 0.086, − 0.058	< 0.001
R^2^	0.173			0.196			0.327		

*ref.* reference, *OR* odds ratio, *CI* confidence interval. Model 1 is adjusted for age, sex, education level, race, Townsend deprivation Index, smoking, drinking, and BMI. Model 2 is adjusted for the covariates in model 1 + NCDs including hypertension, CHD, COPD, diabetes, cataract, asthma, stroke, migraine. Model 3 is adjusted for the covariates in model 2 + depression and physical activity

### Correlations and descriptive analyses of the Main study variables

Sleep quality and duration were negatively correlated with depression (r = − 0.038 and − 0.062, respectively, *P* < 0.001) and positively correlated with HRQoL(r = 0.042 and 0.051, respectively, *P* < 0.001). PA positively correlated with quality of life and negatively correlated with depression(r = 0.040 and − 0.053, respectively, *P* < 0.001). Depression negatively correlated with HRQoL(r = − 0.203, *P* < 0.001) (Table s3).

### Mediation effect analysis between sleep quality and health-related quality of life

**Fig. 3:**
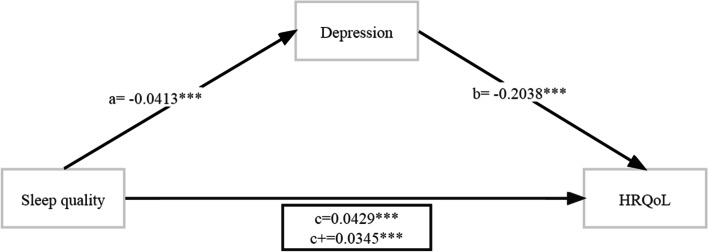
The mediating effect path diagram

Sleep quality had a significant positive predictive effect on HRQoL (β = 0.043; 95% CI, 0.034–0.052), and the direct predictive effect of sleep quality on HRQoL was still significant when depression was added. Meanwhile, sleep quality had a significant negative predictive effect on depression (β = − 0.041; 95% CI, − 0.050 to − 0.030). Depression also had a significant negative predictive effect on HRQoL (β = − 0.204; 95% CI, − 0.215 to − 0.192). Hence, we believe that depression played a partial mediating role (effect = 0.008; 95% CI, 0.007–0.010) in the association between sleep quality and HRQoL, accounting for 19.6% of the total effect (Table s4). The path diagram of the mediation effect is shown in Fig. 3.

### Moderated mediation effect analysis between sleep quality and health-related quality of life

**Fig. 4:**
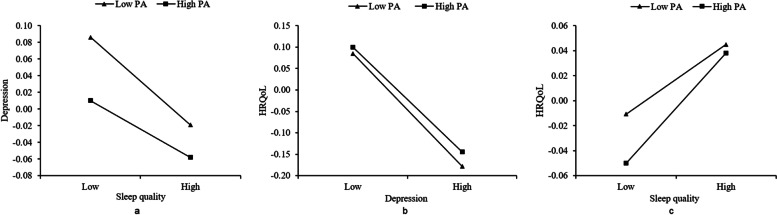
PA moderated the indirect (**a**, **b**) and direct effect (**c**) between sleep quality and HRQoL

After PA was included in the model, the interaction term between sleep quality and PA significantly predicted depression and HRQoL (β = 0.011, *P* = 0.017; β = 0.009, *P* = 0.036), and the interaction term between depression and PA also had a significant predictive effect on HRQoL (β = 0.010, t = 2.149, *P* = 0.022). These findings indicate that PA played a moderating role in the association between sleep quality and HRQoL (Table s5). As PA level increased, the mediating effect of depression on the association between sleep quality and HRQoL declined and the direct effect between sleep quality and HRQoL increased (Table 3). To visualise the moderating effect, PA was divided into a high-level group (M + 1 SD) and a low-level group (M – 1 SD) in simple slope analyses. Sleep quality had a significant predictive effect on depression, but the predictive effect was stronger for the elderly with low-level PA than for those with high-level PA (larger slope) (Fig. 4a). Similarly, PA adversely moderated the mediating effect of depression and positively regulated the direct effect of sleep quality on HRQoL (Fig. 4b and c).

**Table 3:** Effect values of sleep quality on HRQoL at different levels of physical activity

	Physical activities	Effect	Boot SE	Boot LLCI	Boot ULCI
Direct effect	M + 1SD	0.0436	0.0064	0.0313	0.0558
	M	0.0344	0.0044	0.0257	0.0432
	M-1SD	0.0252	0.0066	0.0126	0.0380
	Diff (low and high)	0.0183	0.0096	0.0003	0.0371
Indirect effect	M-1SD	0.0110	0.0015	0.0082	0.0141
	M	0.0084	0.0059	0.0065	0.0102
	M + 1SD	0.0059	0.0012	0.0035	0.0083
	Diff (low and high)	−0.0051	0.0020	−0.0090	−0.0014

Standardized variables were substituted into the regression equation; *M* mean, *SD* standard deviation, *Diff* difference, *SE* standard error, *LLCI* lower limit confidence interval, *ULCI* Upper limit confidence interval

### Quadratic sleep duration on depression and health-related quality of life

After the quadratic term of sleep duration was included in the model (model b), R^2^ increased significantly, indicating a significant curvilinear association among sleep duration, depression, and HRQoL (Table s6). After visualising this nonlinear association, we found a significant U-shaped association between sleep duration and depression and sleep duration and HRQoL (Fig. s5).

### Mediation effect analysis between sleep duration and health-related quality of life

**Fig. 5:**
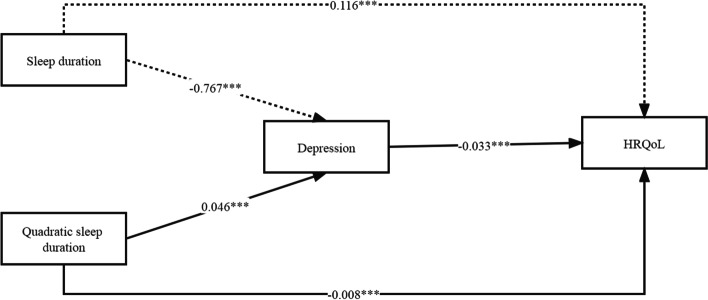
The mediation model of quadratic sleep duration on HRQoL through depression among the elderly

The mediation model is presented in Fig. 5, and the path results are presented in Table s7. In this model, quadratic sleep duration directly affected depression (β = 0.046, *P* < 0.001) and HRQoL (β = − 0.008, *P* < 0.001), whereas depression directly affected HRQoL (β = − 0.033, *P* < 0.001). The results showed that depression partially mediated the quadratic association between sleep duration and HRQoL. The values of the instantaneous indirect effect (θ) indicated that sleep duration positively affected HRQoL through depression among the elderly with short and moderate sleep duration (β = 0.004; 95% CI, 0.004–0.005; β = 0.001; 95% CI, 0.001–0.002) and that the mediating effect of depression decreased with the prolongation of sleep duration (from low to moderate). Conversely, when the elderly had a long sleep duration, sleep duration negatively affected HRQoL through depression (β = − 0.002; 95% CI, − 0.002 to − 0.001).

### Moderated mediation effect analysis between sleep duration and health-related quality of life

**Fig. 6:**
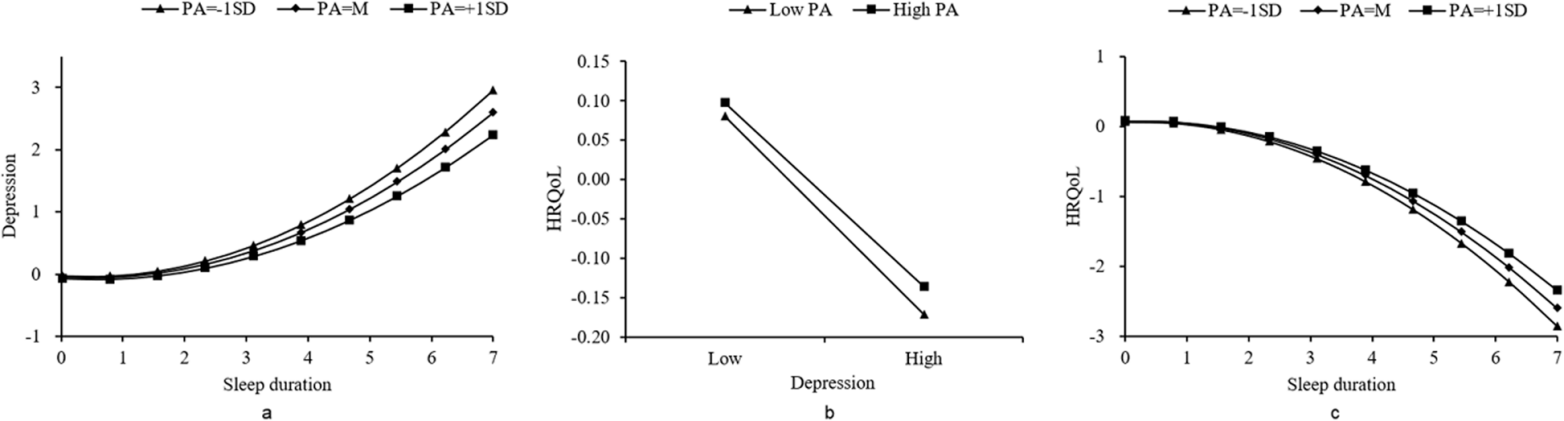
PA moderated the mediating (**a**, **b**) and direct effect (**c**) between sleep duration and HRQoL

The effects of the interaction term of quadratic sleep duration and PA on depression and HRQoL were statistically significant (β = − 0.008, *P* = 0.001; β = 0.006, *P* = 0.017). The results indicated that PA regulated the nonlinear association between sleep duration and depression and sleep duration and HRQoL (Table 4). Simple slope analysis showed that when PA levels increased from low to high, the curvilinear association between sleep duration and depression blunted (the curve was flatter) (Fig. 6a). Similarly, PA moderated the effects of sleep duration and depression on HRQoL (Fig. 6b and c). Specifically, the higher the PA level, the weaker the direct and indirect effects of sleep duration on the quality of life (Table s8).

**Table 4:** Summary of moderated mediation model results between the sleep duration and HRQoL among the elderly

	Depression				HRQoL			
	β	SE	*P*	95%CI	β	SE	*P*	95%CI
Linear sleep duration	−0.0561	0.0045	< 0.001	−0.0648, − 0.0473	0.0298	0.0044	< 0.001	0.0213, 0.0384
Quadratic sleep duration	0.0576	0.0026	< 0.001	0.0525, 0.0628	−0.0573	0.0026	< 0.001	−0.0623, − 0.0523
PA	−0.0259	0.0051	< 0.001	−0.0360, − 0.0158	0.0082	0.0050	0.103	−0.0016, 0.0180
Linear sleep duration *PA	−0.0034	0.0043	0.432	−0.0118, 0.0051	0.0044	0.0042	0.292	−0.0038, 0.0127
Quadratic sleep duration *PA	−0.0080	0.0024	0.001	−0.0127, − 0.0034	0.0056	0.0023	0.017	0.0010. 0.0101
Depression					−0.1927	0.0044	< 0.001	−0.2013, − 0.1841
Depression* PA					0.0088	0.0044	0.046	0.0002, 0.0175
R^2^(F, *P*)	0.0532(*F* = 471.4726, *P* < 0.001)				0.0141(*F* = 142.5452, *P* < 0.001)			

*Sd* sleep duration, *PA* physical activity, *HRQoL* health-related quality of life, *SE* standard error, *CI* confidence interval

## Discussion

To the best of our knowledge, this study is the first to explore the association between sleep disorders and HRQoL among elderly individuals in the United Kingdom using a large nationally representative sample from the UKB database. Moreover, we first used a moderated mediation analysis to elucidate the mediating role of depression between sleep quality and duration and HRQoL and the moderating role of PA among them. Compared with previous epidemiological studies that mainly focused on HRQoL among patients with sleep disorders, our study not only confirmed the negative effect of sleep disorders on HRQoL but also further clarified the underlying path mechanism of sleep disorders leading to low HRQoL. These findings are expected to contribute to the development of targeted interventions to improve HRQoL among elderly individuals in the United Kingdom.

The association between sleep disorders and HRQoL among the elderly has been widely explored in China [14, 16], Spain [17], and other countries [18, 19]. However, due to differences in race, sample size, HRQoL instruments selected, and potential confounders, our study is slightly different from them. For example, a study based on 5539 elderly Chinese community participants found no significant association between sleep duration and HRQoL utility score [16]. Conversely, our findings indicated that short or long sleep duration was significantly negatively correlated with HRQoL utility score, which may be attributed to the differences of sleep patterns among different ethnic groups. A previous Spanish study found that the association between sleep duration and HRQoL was much stronger than our study [17]. Nevertheless, there is evidence that sleep quality is more strongly associated with HRQoL than sleep duration [21]. Hence, the difference may be subject to the fact that the study did not include sleep quality, resulting in an overestimation of the study effect. Accordingly, a large number of well-designed UKB data, including more detailed sleep duration and a novel sleep quality score, are warranted to validate the association. Obviously, determining effective interventions that directly regulate sleep disorders has proven to be challenging. Therefore, exploring alternative approaches to reduce the adverse effects of sleep disorders on HRQoL is crucial for improving HRQoL in the elderly.

In this study, we found that depression acts as a partial mediator in the relationships between sleep disorders and HRQoL. Previous studies regarding sleep disorders were significant predictors of depression in the elderly, supporting our findings [47–54]. One possible explanation was that the elderly with sleep disorders were often accompanied by reduced cognition, physical function, and vitality and poor subjective health [2, 17, 48, 55], all of which are significant precursors of depression [56]. Additionally, sleep disorders can lead to a range of adverse consequences, such as poor concentration and reduced glucose tolerance and sympathetic nervous system activation, which can trigger depressive symptoms [14]. Furthermore, depression, which has been widely documented, is associated with chronic disease incidence, comorbidity, and mortality, ultimately leading to a decline in the HRQoL of the elderly [40, 56–58]. Many empirical studies have indicated that poor mental health, mainly depression, could have a serious negative effect on HRQoL among the elderly [2, 59, 60] These results suggest that targeted interventions for depression disorders may mitigate the effect of sleep disorders on impaired HRQoL in the elderly.

PA, an easily regulated low-cost behavioural factor, was found to have a moderating effect on both the adverse effect of sleep disorders on HRQoL and on the mediating effects of depression based on a moderated mediation analysis. In other words, although sleep disorders, either directly or indirectly through depression, had a negative effect on HRQoL, the elderly who experienced high PA levels were less affected. Our results were consistent with the previous findings that PA could exert a positive influence on sleep disorders and depression [29, 61, 62]. For example, one study suggested that PA can improve sleep by improving depression symptoms in adults aged above 60 years [62] and ultimately improve their health. Another clinical trial found that inactive PA worsens sleep quality and mental health of patients with insomnia, further reducing their HRQoL [29]. Additionally, a study in Southeast Asia suggested that PA can prevent depression and improve sleep in the elderly [61]. BDNF is a neurotrophic factor expressed in the hippocampus and is thought to play a key role in depression [63]. Mechanism studies have shown that PA significantly increases the expression of BDNF in the brain, and increased PA may actively promote brain growth, especially in the hippocampus [64]. Hence, it is reasonable to believe that high PA levels may mitigate some of the adverse effects of depression-mediated sleep disorders on HRQoL. Meanwhile, our findings showed that sleep disorders had a greater effect on HRQoL among the elderly with low-level PA than high-level PA, which indicated that PA moderated the independent association between sleep disorders and HRQoL. One proposed physiological mechanism suggested that PA increases body temperature, thereby promoting sleep by activating the cooling process and sleep-inducing mechanisms controlled by the hypothalamus, and that increased energy expenditure also leads to increased non-rapid eye movement sleep, based on the energy conservation theory [65]. A previous study also discovered that moderate-intensity aerobic exercise, such as bikes or treadmills, improved self-reported sleep, mood, and HRQoL in elderly individuals with chronic insomnia [28]. Evidence has shown that sleep disorders may lead to reduced maximal oxygen uptake, increased exercise-related injuries, daytime fatigue, and reduced regular participation in PA, increasing the risk of adverse health outcomes and ultimately worsening HRQoL in the elderly [66].

### Study strengths and limitations

The present study has several strengths. First, the findings are highly representative of the elderly in the United Kingdom as the study included a nationally representative large sample size of participants and adjusted for potential confounding factors. Second, the novel sleep quality score, which combines various basic sleep characteristics, provides a convenient and comprehensive method for studying the complex effects of sleep and other time-dependent variables. Finally, this study adds new knowledge to the examination of mediating and moderated mediating effects in the nonlinear association between sleep duration and HRQoL.

This study has some limitations. First of all, in UK Biobank, the low response rate (5.5%) affected by selection bias [67] and the study sample being mostly of European descent may influence the generality of our results extrapolating to other populations. However, studies have showed that the risk factor associations in the UK Biobank appear to be universal despite the low response rate [68]. Secondly, dichotomising various factors may lead to loss of information and statistical power in multivariate analysis. However, the classification treatment of various continuous variables in current study is not only based on the conventional practice of previous literature [39, 69], but also makes the interpretation and presentation of results easier to understand [70]. In addition, the sleep duration variable collected in this study was self-reported information, which may lead to recall bias. Nevertheless, there is a good correlation between subjective sleep duration and sleep duration measured using objective tools [71]. Finally, depression only partially mediates the association between sleep disorders and HRQoL, which indicates that exploring other mediating factors among the association in the elderly is warranted in the future.

## Conclusions

Our study showed that poor sleep quality and abnormal sleep duration were associated with impaired HRQoL among elderly individuals in the United Kingdom. Depression partially mediated this association. Additionally, PA moderated all paths among sleep, depression, and HRQoL, and greater effects were observed in the elderly with lower PA levels. Hence, we suggest that healthcare decision-makers should regularly monitor the mental health of the elderly, especially those with sleep disorders, and encourage them to participate in physical activities as much as possible to prevent or alleviate the negative effects of depression and ultimately improve their HRQoL.

## Supplementary Information

**Additional file 1.**

## Data Availability

The data used in current study belongs to Soochow University. According to Soochow University MEDICAL Ethics Board regulations, these data will not be shared online, but can be obtained from the corresponding author of this study upon reasonable request.

## References

[1] Grandner MA (2020). Sleep, health, and society. Sleep Med Clin.

[2] Yuan Y, Li J, Jing Z, Yu C, Zhao D, Hao W, Zhou C (2020). The role of mental health and physical activity in the association between sleep quality and quality of life among rural elderly in China: a moderated mediation model. J Affect Disord.

[3] Patte KA, Qian W, Leatherdale ST (2017). Sleep duration trends and trajectories among youth in the COMPASS study. Sleep Health.

[4] Kryger MH, Roth T: Principles and practice of sleep medicine. In: Elsevier; 2017.

[5] Crowley K (2011). Sleep and sleep disorders in older adults. Neuropsychol Rev.

[6] LeBlanc M, Mérette C, Savard J, Ivers H, Baillargeon L, Morin CM (2009). Incidence and risk factors of insomnia in a population-based sample. Sleep.

[7] Allen RP, Walters AS, Montplaisir J, Hening W, Myers A, Bell TJ, Ferini-Strambi L (2005). Restless legs syndrome prevalence and impact: REST general population study. Arch Intern Med.

[8] Chen YL, Weng SF, Shen YC, Chou CW, Yang CY, Wang JJ, Tien KJ (2014). Obstructive sleep apnea and risk of osteoporosis: a population-based cohort study in Taiwan. J Clin Endocrinol Metab.

[9] Dew MA, Hoch CC, Buysse DJ, Monk TH, Begley AE, Houck PR, Hall M, Kupfer DJ, Reynolds CF (2003). Healthy older adults' sleep predicts all-cause mortality at 4 to 19 years of follow-up. Psychosom Med.

[10] Kay DB, Dzierzewski JM (2015). Sleep in the context of healthy aging and psychiatric syndromes. Sleep Med Clin.

[11] Sun XH, Ma T, Yao S, Chen ZK, Xu WD, Jiang XY, Wang XF (2020). Associations of sleep quality and sleep duration with frailty and pre-frailty in an elderly population Rugao longevity and ageing study. BMC Geriatr.

[12] Ohayon MM, Vecchierini MF (2005). Normative sleep data, cognitive function and daily living activities in older adults in the community. Sleep.

[13] Cho J, Kwak N, Choi SM, Lee J, Park YS, Lee CH, Lee SM, Yoo CG, Kim YW, Han SK: Sleep duration and health-related quality of life in Korean adults: 2007-2015 Korea National Health and nutrition examination survey. Sleep Breathing Schlaf Atmung 2020, 24(2):725–733. 10.1007/s11325-019-01972-7 31792907

[14] Lo CM, Lee PH (2012). Prevalence and impacts of poor sleep on quality of life and associated factors of good sleepers in a sample of older Chinese adults. Health Qual Life Outcomes.

[15] Liu Y, Dong Y, Li X, Mao X, Peng G, Liu LJMPM: Meta-analysis of the prevalence of sleep disorder among Chinese elderly aged 60 years and over. 2014, 41(8):1442–1445.

[16] Pan CW, Cong X, Zhou HJ, Li J, Sun HP, Xu Y, Wang P (2017). Self-reported sleep quality, duration, and health-related quality of life in older Chinese: evidence from a rural town in Suzhou, China. J Clin Sleep Med.

[17] Faubel R, Lopez-Garcia E, Guallar-Castillón P, Balboa-Castillo T, Gutiérrez-Fisac JL, Banegas JR, Rodríguez-Artalejo F (2009). Sleep duration and health-related quality of life among older adults: a population-based cohort in Spain. Sleep.

[18] Nogueira B, Li L, Meng LR, Ungvari GS, Forester BP, Chiu HFK, Kuok KCF, Tran L, Liu ZM, Xiang YT (2018). Prevalence of sleep disturbances and their associations with demographic and clinical characteristics and quality of life in older adults in Macao. Perspectives Psychiatric Care.

[19] Fagerström C, Hellström A (2011). Sleep complaints and their association with comorbidity and health-related quality of life in an older population in Sweden. Aging Ment Health.

[20] Jean-Louis G, Kripke DF, Ancoli-Israel S (2000). Sleep and quality of well-being. Sleep.

[21] Pilcher JJ, Ginter DR, Sadowsky B (1997). Sleep quality versus sleep quantity: relationships between sleep and measures of health, well-being and sleepiness in college students. J Psychosom Res.

[22] Ge Y, Xin S, Luan D, Zou Z, Liu M, Bai X, Gao Q (2019). Association of physical activity, sedentary time, and sleep duration on the health-related quality of life of college students in Northeast China. Health Qual Life Outcomes.

[23] Liu X, Wang C, Qiao X, Si H, Jin Y: Sleep quality, depression and frailty among Chinese community-dwelling older adults. Geriatric Nurs. 2021, 42(3):714–720. 10.1016/j.gerinurse.2021.02.020 33836251

[24] van Mill JG, Vogelzangs N, van Someren EJ, Hoogendijk WJ, Penninx BW (2014). Sleep duration, but not insomnia, predicts the 2-year course of depressive and anxiety disorders. J Clin Psychiatry.

[25] Ghimire S, Baral BK, Pokhrel BR, Pokhrel A, Acharya A, Amatya D, Amatya P, Mishra SR (2018). Depression, malnutrition, and health-related quality of life among Nepali older patients. BMC Geriatr.

[26] Rhee TG, Steffens DC (2020). Major depressive disorder and impaired health-related quality of life among US older adults. Int J Geriatric Psychiatry.

[27] Cuijpers P, Karyotaki E, Eckshtain D, Ng MY, Corteselli KA, Noma H, Quero S, Weisz JR (2020). Psychotherapy for depression across different age groups: a systematic review and Meta-analysis. JAMA Psychiatry.

[28] Reid KJ, Baron KG, Lu B, Naylor E, Wolfe L, Zee PC (2010). Aerobic exercise improves self-reported sleep and quality of life in older adults with insomnia. Sleep Med.

[29] Hartescu I, Morgan K, Stevinson CD (2015). Increased physical activity improves sleep and mood outcomes in inactive people with insomnia: a randomized controlled trial. J Sleep Res.

[30] Knapen J, Vancampfort D, Moriën Y, Marchal Y (2015). Exercise therapy improves both mental and physical health in patients with major depression. Disabil Rehabil.

[31] Sudlow C, Gallacher J, Allen N, Beral V, Burton P, Danesh J, Downey P, Elliott P, Green J, Landray M (2015). UK biobank: an open access resource for identifying the causes of a wide range of complex diseases of middle and old age. PLoS Med.

[32] Lombardi DA, Folkard S, Willetts JL, Smith GS (2010). Daily sleep, weekly working hours, and risk of work-related injury: US National Health Interview Survey (2004-2008). Chronobiol Int.

[33] Daghlas I, Dashti HS, Lane J, Aragam KG, Rutter MK, Saxena R, Vetter C (2019). Sleep duration and myocardial infarction. J Am Coll Cardiol.

[34] Fan M, Sun D, Zhou T, Heianza Y, Lv J, Li L, Qi L (2020). Sleep patterns, genetic susceptibility, and incident cardiovascular disease: a prospective study of 385 292 UK biobank participants. Eur Heart J.

[35] Helou K, El Helou N, Mahfouz M, Mahfouz Y, Salameh P, Harmouche-Karaki M (2017). Validity and reliability of an adapted arabic version of the long international physical activity questionnaire. BMC Public Health.

[36] Kroenke K, Spitzer RL, Williams JB (2003). The patient health Questionnaire-2: validity of a two-item depression screener. Med Care.

[37] Wong EL, Yeoh EK, Slaap B, Tam WW, Cheung AW, Wong AY, Chan DCJViH: PRM101 Validation And Valuation Of The Preference-Based Healthindex Using Eq-5d-5l In The Hong Kong Population. 2015, 18(3):A27.

[38] Devlin NJ, Shah KK, Feng Y, Mulhern B, van Hout B (2018). Valuing health-related quality of life: an EQ-5D-5L value set for England. Health Econ.

[39] Sung SA, Hyun YY, Lee KB, Park HC, Chung W, Kim YH, Kim YS, Park SK, Oh KH, Ahn C (2018). Sleep duration and health-related quality of life in Predialysis CKD. Clin J Am Soc Nephrol.

[40] Dregan A, Rayner L, Davis KAS, Bakolis I (2020). Arias de la Torre J, das-Munshi J, hatch SL, Stewart R, Hotopf M: associations between depression, arterial stiffness, and metabolic syndrome among adults in the UK biobank population study: a mediation analysis. JAMA Psychiatry.

[41] Austin PC, Escobar M, Kopec JA (2000). The use of the Tobit model for analyzing measures of health status. Quality Life Res.

[42] Hayes A, Rockwood NJJBR, Therapy: Regression-based statistical mediation and moderation analysis in clinical research: Observations, recommendations, and implementation 2016:39. 10.1016/j.brat.2016.11.001 27865431

[43] Hayes AF, Preacher KJ (2010). Quantifying and testing indirect effects in simple mediation models when the constituent paths are nonlinear. Multivar Behav Res.

[44] Hayes AF, Scharkow M (2013). The relative trustworthiness of inferential tests of the indirect effect in statistical mediation analysis: does method really matter?. Psychol Sci.

[45] Hao W, Li J, Fu P, Zhao D, Jing Z, Wang Y, Yu C, Yuan Y, Zhou C (2021). Physical frailty and health-related quality of life among Chinese rural older adults: a moderated mediation analysis of physical disability and physical activity. BMJ Open.

[46] Hayes AF. Hacking PROCESS for estimation and probing of linear moderation of quadratic effects and quadratic moderation of linear effects. Unpublished White Paper). Ohio State University; 2015.

[47] Fang H, Tu S, Sheng J, Shao A (2019). Depression in sleep disturbance: a review on a bidirectional relationship, mechanisms and treatment. J Cell Mol Med.

[48] Thomas KM, Redd LA, Wright JD, Hartos JL (2017). Sleep and mental health in the general population of elderly women. J Prim Prev.

[49] Fang B, Liu H, Yang S, Xu R, Chen G (2020). Sleep duration, depression, and peptic ulcer recurrence in older patients with mild cognitive impairment. Health Psychol.

[50] Guan Q, Hu X, Ma N, He H, Duan F, Li X, Luo Y, Zhang H (2020). Sleep quality, depression, and cognitive function in non-demented older adults. J Alzheimer's Dis.

[51] Yu J, Rawtaer I, Fam J, Jiang MJ, Feng L, Kua EH, Mahendran R (2016). Sleep correlates of depression and anxiety in an elderly Asian population. Psychogeriatrics.

[52] Hoyos CM, Gordon C, Terpening Z, Norrie L, Lewis SJG, Hickie IB, Naismith SL (2020). Circadian rhythm and sleep alterations in older people with lifetime depression: a case-control study. BMC psychiatry.

[53] Paudel ML, Taylor BC, Diem SJ, Stone KL, Ancoli-Israel S, Redline S, Ensrud KE (2008). Association between depressive symptoms and sleep disturbances in community-dwelling older men. J Am Geriatr Soc.

[54] Wolkove N, Elkholy O, Baltzan M, Palayew M: Sleep and aging: 1. Sleep disorders commonly found in older people. Can Med Assoc J. 2007, 176(9):1299–1304. 10.1503/cmaj.060792 PMC1852874 17452665

[55] Chen Q, Hayman LL, Shmerling RH, Bean JF, Leveille SG (2011). Characteristics of chronic pain associated with sleep difficulty in older adults: the maintenance of balance, independent living, intellect, and zest in the elderly (MOBILIZE) Boston study. J Am Geriatr Soc.

[56] Hammen C (2018). Risk factors for depression: an autobiographical review. Annu Rev Clin Psychol.

[57] Liu X, Cao H, Zhu H, Zhang H, Niu K, Tang N, Cui Z, Pan L, Yao C, Gao Q (2021). Association of chronic diseases with depression, anxiety and stress in Chinese general population: the CHCN-BTH cohort study. J Affect Disord.

[58] Gerino E, Rollè L, Sechi C, Brustia P (2017). Loneliness, resilience, mental health, and quality of life in old age: a structural equation model. Front Psychol.

[59] Blazer DG (2003). Depression in late life: review and commentary. J Gerontol A Biol Sci Med Sci.

[60] Xie JF, Ding SQ, Zhong ZQ, Yi QF, Zeng SN, Hu JH, Zhou JD (2014). Mental health is the most important factor influencing quality of life in elderly left behind when families migrate out of rural China. Revista Latino-Americana de Enfermagem.

[61] Kadariya S, Gautam R, Aro AR (2019). Physical activity, mental health, and wellbeing among older adults in south and Southeast Asia: a scoping review. Biomed Res Int.

[62] Montgomery P, Dennis J: Physical exercise for sleep problems in adults aged 60+. Cochrane Database Syst Rev. 2002, 2002(4):Cd003404. 10.1002/14651858.CD003404 PMC7017641 12519595

[63] von Bohlen Und Halbach O, von Bohlen Und Halbach V: BDNF effects on dendritic spine morphology and hippocampal function. Cell Tissue Res 2018, 373(3):729–741. 10.1007/s00441-017-2782-x 29450725

[64] Mattson MP (2012). Evolutionary aspects of human exercise--born to run purposefully. Ageing Res Rev.

[65] Andrade FM, Pedrosa RP (2016). The role of physical exercise in obstructive sleep apnea. J Brasileiro de Pneumologia.

[66] Štefan L, Vrgoč G, Rupčić T, Sporiš G, Sekulić D. Sleep duration and sleep quality are associated with physical activity in elderly people living in nursing homes. Int J Environ Res Public Health. 2018;15(11):2512. 10.3390/ijerph15112512 PMC6266288 30423981

[67] Swanson JM: The UK Biobank and selection bias. Lancet (London, England) 2012, 380(9837):110. 10.1016/S0140-6736(12)61179-9 22794246

[68] Batty GD, Gale CR, Kivimäki M, Deary IJ, Bell S: Comparison of risk factor associations in UK Biobank against representative, general population based studies with conventional response rates: prospective cohort study and individual participant meta-analysis. BMJ (Clinical research ed) 2020, 368:m131. 10.1136/bmj.m131 PMC7190071 32051121

[69] Sambou ML, Zhao X, Hong T, Fan J, Basnet TB, Zhu M, Wang C, Hang D, Jiang Y, Dai J (2021). Associations between sleep quality and health span: a prospective cohort study based on 328,850 UK biobank participants. Front Genet.

[70] Altman DG, Royston P: The cost of dichotomising continuous variables. BMJ (Clinical research ed) 2006, 332(7549):1080. 10.1136/bmj.332.7549.1080 PMC1458573 16675816

[71] Lockley SW, Skene DJ, Arendt J (1999). Comparison between subjective and actigraphic measurement of sleep and sleep rhythms. J Sleep Res.

