# Supplementary material for: The role of depression and physical activity in the association of between sleep quality, and duration with and health-related quality of life among the elderly: a UK Biobank cross-sectional study
Source: BMC Geriatr. 2022 Apr 19;22:338. doi: 10.1186/s12877-022-03047-x (PMC9016983; doi:10.1186/s12877-022-03047-x)
Supplement: Supplementary file 1 — Additional file 1. [file 12877_2022_3047_MOESM1_ESM.docx]

**Supplementary Info**

Table s1. An EQ-5D-5L value set for England

| Levels of five dimensions | Estimate |
| --- | --- |
| Constant | 1.000 |
| Mobility |  |
| No problems | 0 |
| Slight problems | 0.058 |
| Moderate problems | 0.076 |
| Severe problems | 0.207 |
| Unable | 0.274 |
| Self-care |  |
| No problems | 0 |
| Slight problems | 0.050 |
| Moderate problems | 0.080 |
| Severe problems | 0.164 |
| Unable | 0.203 |
| Usual activities |  |
| No problems | 0 |
| Slight problems | 0.050 |
| Moderate problems | 0.063 |
| Severe problems | 0.162 |
| Unable | 0.184 |
| Pain/discomfort |  |
| No problems | 0 |
| Slight problems | 0.063 |
| Moderate problems | 0.084 |
| Severe problems | 0.276 |
| Extreme problems | 0.335 |
| Anxiety/depression |  |
| No problems | 0 |
| Slight problems | 0.078 |
| Moderate problems | 0.104 |
| Severe problems | 0.285 |
| Extreme problems | 0.289 |

Table s2. Characteristics of study participants according to sleep duration of the elderly in the UK

| Baseline characteristics | <=5 | 6 | 7-8 | 9 | >=10 | *P* |
| --- | --- | --- | --- | --- | --- | --- |
|  | (n=1868) | (n=8702) | (n=36870) | (n=3464) | (n=647) |  |
| Sociodemographic characteristics |  |  |  |  |  |  |
| Age, years | 63.7 ± 2.8 | 63.4 ± 2.7 | 63.6 ± 2.7 | 64.0 ± 2.8 | 64.0 ± 2.9 | <0.001 |
| Female | 1095(58.6) | 4584(52.7) | 18347(49.8) | 1691(48.8) | 295(45.6) | <0.001 |
| College or university degree | 563(30.1) | 3415 (39.2) | 15844(43.0) | 1278(36.9) | 194(30.0) | <0.001 |
| Townsend Index | -1.5 ± 2.9 | -1.8 ± 2.8 | -2.0 ± 2.7 | -2.0 ± 2.6 | -1.5 ± 3.0 | <0.001 |
| Ethnicity, white race | 1796(96.6) | 8488(97.9) | 36277(98.7) | 3412(99.0) | 626(97.4) | <0.001 |
| BMI, kg/m^2^ | 27.5 ± 4.7 | 27.1 ± 4.4 | 26.7 ± 4.1 | 27.3 ± 4.2 | 28.3 ± 5.0 | <0.001 |
| Sum MET, min | 2790 ± 2811 | 2633 ± 2572 | 2595 ± 2421 | 2525 ± 2382 | 2376 ± 2469 | <0.001 |
| Depression score | 0.6 ± 1.1 | 0.4 ± 0.9 | 0.3 ± 0.7 | 0.3 ± 0.8 | 0.6 ± 1.2 | <0.001 |
| Current or former smoker | 937(50.2) | 4216(48.5) | 17214(46.7) | 1698(49.0) | 345(53.3) | <0.001 |
| Current or former alcohol drinker | 1780(95.3) | 8391(96.4) | 35845(97.2) | 3339(96.4) | 619(95.7) | <0.001 |
| NCDs |  |  |  |  |  |  |
| Hypertension | 286(15.3) | 1089(12.5) | 3985(10.8) | 498(14.4) | 120(18.6) | <0.001 |
| Diabetes | 112(6.0) | 426(4.9) | 1516(4.1) | 225(6.5) | 71(11.0) | <0.001 |
| Cataract | 81(4.3) | 392(4.5) | 1610(4.4) | 181(5.2) | 47(7.3) | 0.002 |
| Stroke | 56(3.0) | 186(2.1) | 728(2.0) | 96(2.8) | 27(4.2) | <0.001 |
| CHD | 158(8.5) | 529(6.1) | 2083(5.7) | 260(7.5) | 68(10.5) | <0.001 |
| COPD | 51(2.8) | 190(2.2) | 638(1.7) | 73(2.1) | 17(2.6) | 0.001 |
| Asthma | 192(10.6) | 744(8.8) | 2603(7.3) | 234(7.0) | 72(11.5) | <0.001 |
| Migraine | 82(4.4) | 324(3.7) | 1156(3.2) | 110(3.2) | 29(4.5) | 0.001 |
| Low-risk sleep factors |  |  |  |  |  |  |
| Early chronotype | 1043(62.1) | 4837(62.0) | 20708(62.5) | 1916(61.6) | 349(59.1) | 0.364 |
| Sleep 7–8 h/day | - | - | - | - | - |  |
| Never/rarely insomnia | 478(25.6) | 2222(25.5) | 9487(25.7) | 904(26.1) | 138(21.3) | 0.142 |
| No self-reported snoring | 1116(62.7) | 5292(64.2) | 22373(64.4) | 2083(63.7) | 387(64.2) | 0.617 |
| No frequent daytime sleepiness | 1835(98.3) | 8505(97.9) | 36010(97.8) | 3392(98.0) | 639(98.8) | 0.182 |
| EQ-5D-5L |  |  |  |  |  |  |
| Utility score | 0.829 ± 0.168 | 0.870 ± 0.142 | 0.891 ± 0.127 | 0.875 ± 0.144 | 0.813 ± 0.201 | <0.001 |
| Problems of Dimensions |  |  |  |  |  |  |
| Mobility | 904(48.4) | 3219(37.0) | 12139(32.9) | 1276(36.8) | 328(50.7) | <0.001 |
| Self-care | 330(17.7) | 1035(11.9) | 3456(9.4) | 421(12.2) | 134(20.7) | <0.001 |
| Usual activities | 910(48.7) | 3495(40.2) | 12900(35.0) | 1346(38.9) | 342(52.9) | <0.001 |
| Pain/discomfort | 1306(69.9) | 5362(61.6) | 20497(55.6) | 1970(56.9) | 419(64.8) | <0.001 |
| Anxiety/depression | 595(31.9) | 2225(25.6) | 7339(19.9) | 805(23.2) | 204(31.5) | <0.001 |

Note, EQ-5D-5L, European Quality of Life-5 Dimensions 5-levels; CHD, coronary heart disease; COPD, chronic obstructive pulmonary disease. Continuous variables presented as mean ± SD (standard deviation) and categorical variables presented as n (%).

Table s3. Descriptive statistics and Pearson correlation analysis results of study variable

| Variable | 1 | 2 | 3 | 4 | 5 |
| --- | --- | --- | --- | --- | --- |
| 1 (Sleep duration) | 1 |  |  |  |  |
| 2 (Sleep quality) | 0.166*** | 1 |  |  |  |
| 3 (PA) | 0.003 | 0.006 | 1 |  |  |
| 4 (Depression) | -0.062*** | -0.038*** | -0.053*** | 1 |  |
| 5 (HRQoL) | 0.051*** | 0.042*** | 0.040*** | -0.203*** 0.203***0.203***0.203*** | 1 |
| Mean | 7.253 | 3.115 | 2601 | 0.325 | 0.883 |
| SD | 1.003 | 0.951 | 2461 | 0.795 | 0.135 |

Note, PA, physical activity; SD, standard deviation; HRQoL, health-related quality of life; ****P*<0.001.

Table s4. Mediating model test of depression between sleep quality and HRQoL of elderly in the UK.

| Process | R^2^ | F | Effect | Boot SE | Boot LLCI | Boot ULCI |
| --- | --- | --- | --- | --- | --- | --- |
| Sleep quality to HRQoL: | 0.002 | 93.075^***^ |  |  |  |  |
| Total effect (path c) |  |  | 0.0429^***^ | 0.0045 | 0.0342 | 0.0517 |
| Direct effect (path c^+^) |  |  | 0.0345^***^ | 0.0044 | 0.0258 | 0.0428 |
| Mediating effect (path a*b) |  |  | 0.0084^***^ | 0.0010 | 0.0065 | 0.0103 |
| Sleep quality to depression (path a) | 0.002 | 85.223^***^ | -0.0413^***^ | 0.0046 | -0.0501 | -0.0320 |
| Depression to HRQoL (path b) | 0.044 | 1140.726^***^ | -0.2038^***^ | 0.0059 | -0.2154 | -0.1920 |

. *** *P*<0.001.

Note: Standardized variables were substituted into the regression equation; SE, standard error; LLCI, lower limit confidence interval; ULCI, Upper limit confidence interval. Adjusted for the covariates in model 2.

Table s5. Testing the moderated mediation effect of sleep quality on HRQoL of elderly in the UK.

| Predictors | Model 1(Depression) | | | | Model 2(HRQoL) | | | |
| --- | --- | --- | --- | --- | --- | --- | --- | --- |
|  | β | t | *P* | 95%CI | β | t | *P* | 95%CI |
| Sleep quality | -0.041 | -9.232 | <0.001 | -0.050, -0.033 | 0.034 | 7.892 | <0.001 | 0.026, 0.043 |
| PA | -0.033 | -7.418 | <0.001 | -0.042, -0.024 | 0.013 | 2.927 | 0.003 | 0.004, 0.021 |
| SQ*PA | 0.011 | 2.397 | 0.017 | 0.002, 0.020 | 0.009 | 2.102 | 0.036 | 0.002, 0.017 |
| Depression |  |  |  |  | -0.203 | -46.286 | <0.001 | -0.211, -0.194 |
| Depression * PA |  |  |  |  | 0.010 | 2.149 | 0.022 | 0.002, 0.018 |
| R^2^ | 0.003 | | | | 0.044 | | | |
| F(*P*) | 49.116 (<0.001) | | | | 460.074 (<0.001) | | | |

Note, Standardized variables were substituted into the regression equation; SQ, sleep quality; PA, physical activity; CI, confidence interval; HRQoL, health-related quality of life. Adjusted for the covariates in model 2.

Table s6. Linear sleep duration and quadratic sleep duration on depression and HRQoL of the elderly.

|  | Depression | | | | HRQoL | | | |
| --- | --- | --- | --- | --- | --- | --- | --- | --- |
|  | β | t | *P* | R^2^(*P*) | β | t | *P* | R^2^(*P*) |
| Model a |  |  |  | 0.003 (*P*<0.001) |  |  |  | 0.002(*P*<0.001) |
| Intercept | 0.637 | 24.481 | <0.001 |  | 0.845 | 195.436 | <0.001 |  |
| Linear sleep duration | -0.043 | -12.113 | <0.001 |  | 0.005 | 8.828 | <0.001 |  |
| Model b |  |  |  | 0.013(*P*<0.001) |  |  |  | 0.015(*P*<0.001) |
| Intercept | 2.999 | 27.365 | <0.001 |  | 0.375 | 20.672 | <0.001 |  |
| Linear sleep duration | -0.707 | -23.457 | <0.001 |  | 0.137 | 27.521 | <0.001 |  |
| Quadratic sleep duration | 0.046 | 22.183 | <0.001 |  | -0.009 | -26.661 | <0.001 |  |

Table s7. Path results of the mediation models between sleep duration and HRQoL of the elderly.

| Direct effect | β | SE | *P* | R^2^ |
| --- | --- | --- | --- | --- |
| Sleep duration to depression | -0.7067 | 0.0301 | <0.001 |  |
| Quadratic sleep duration to depression | 0.0457 | 0.0021 | <0.001 | 0.013 |
| Sleep duration to HRQoL | 0.1157 | 0.0050 | <0.001 |  |
| Quadratic sleep duration to HRQoL | -0.0077 | 0.0003 | <0.001 | 0.053 |
| Depression to HRQoL | -0.0329 | 0.0007 | <0.001 |  |
| Indirect effect (θ) | β | Boot SE | Bootstrap 95%CI |  |
| M-1SD | 0.0044 | 0.0003 | 0.0039, 0.0050 |  |
| M | 0.0014 | 0.0001 | 0.0011, 0.0017 |  |
| M+1SD | -0.0016 | 0.0003 | -0.0021, -0.0011 |  |

Note: Standardized variables were substituted into the regression equation; M, mean; SD, standard deviation; SE, standard error; CI, confidence interval; HRQoL, health-related quality of life.

Table s8. Effect values of sleep duration on HRQoL at different levels of physical activity

|  | Physical activities | Effect | Boot SE | Boot LLCI | Boot ULCI |
| --- | --- | --- | --- | --- | --- |
| Direct effect | M + 1SD | -0.0518 | 0.0035 | -0.0568 | -0.0449 |
|  | M | -0.0573 | 0.0026 | -0.0623 | -0.0523 |
|  | M - 1SD | -0.0628 | 0.0034 | -0.0695 | -0.0562 |
|  | Diff (low and high) | 0.0110 | 0.0028 | 0.0082 | 0.0114 |
| Indirect effect | M - 1SD | -0.0132 | 0.0012 | -0.0157 | -0.0110 |
|  | M | -0.0111 | 0.0009 | -0.0129 | -0.0095 |
|  | M + 1SD | -0.0091 | 0.0011 | -0.0114 | -0.0071 |
|  | Diff (low and high) | 0.0041 | 0.0015 | 0.0011 | 0.0071 |

Note: Standardized variables were substituted into the regression equation; M, mean; SD, standard deviation; Diff, difference; SE, standard error; HRQoL, health-related quality of life; LLCI, lower limit confidence interval; ULCI, Upper limit confidence interval.


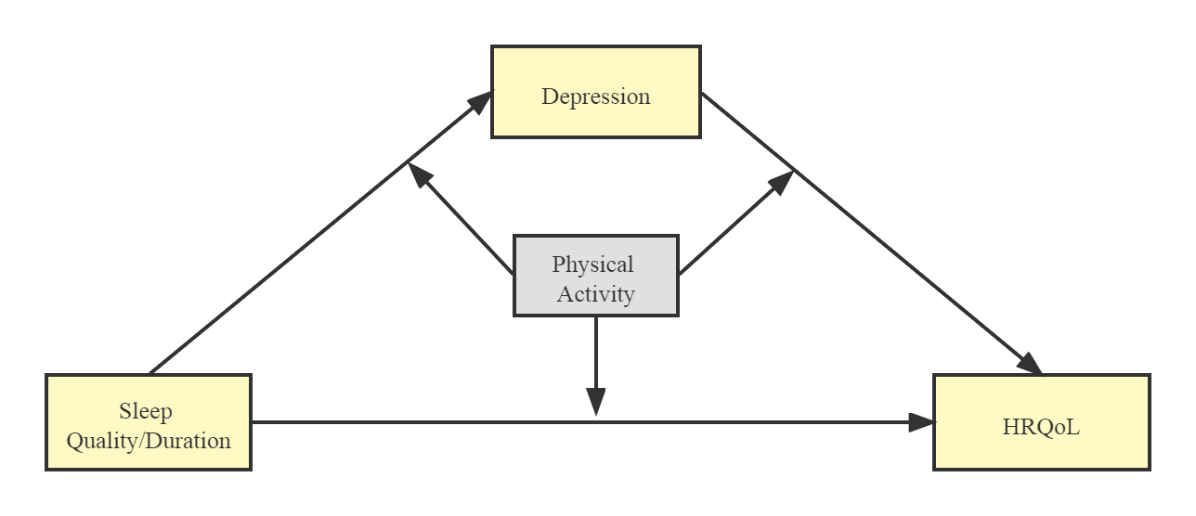
 Figure s1. Theoretical model: a moderated mediation model.


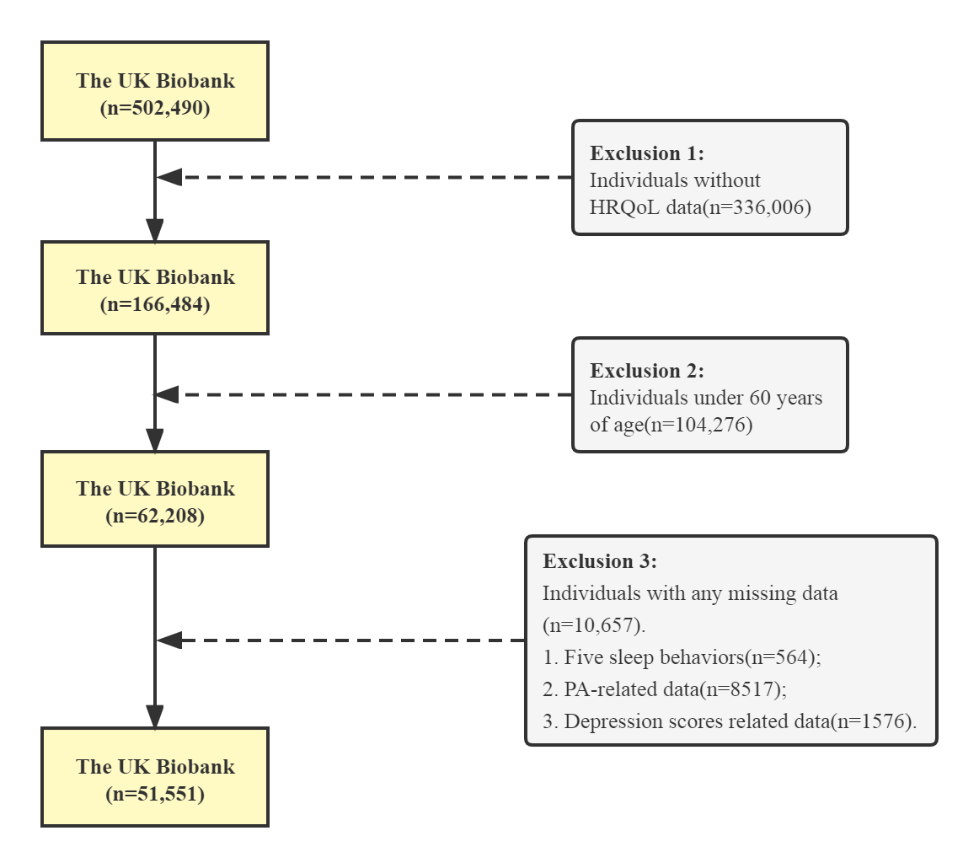


Figure s2. The selection process for the study sample among the elderly from the UK Biobank database.


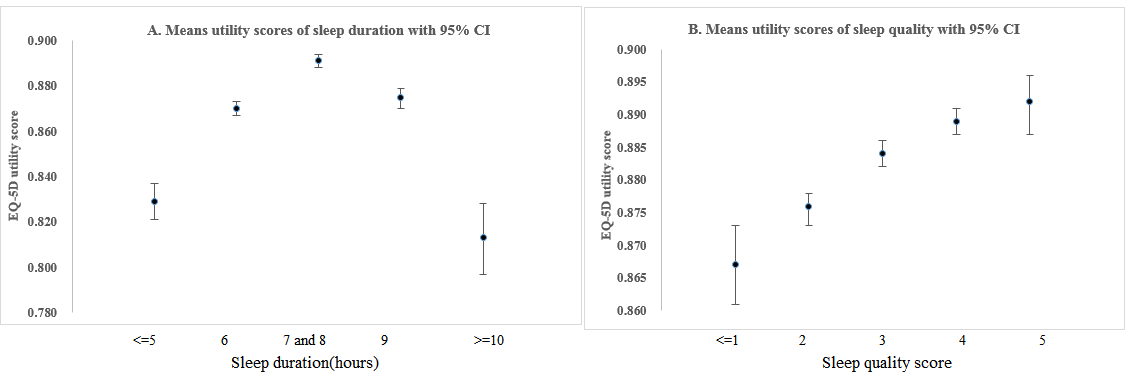


Figure s3. Mean EQ-5D-5L utility scores (95% CI) according to sleep duration (A) and quality (B).

Note, EQ-5D-5L, European Quality of Life-5 Dimensions 5-levels; CI, confidence interval.


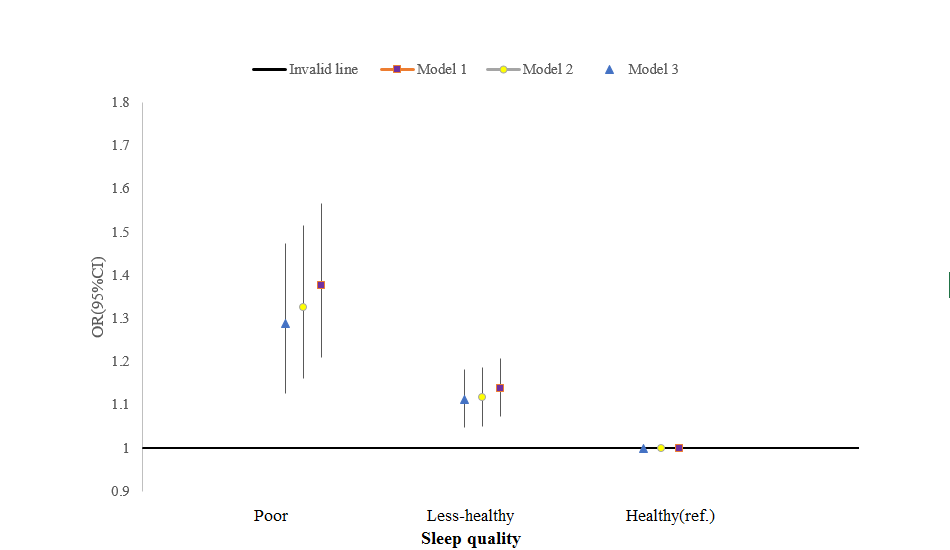


Figure s4. Association between sleep quality categories and low health-related quality of life among the elderly.

Note, ref, reference; OR, odds ratio; CI, confidence interval. Model 1 is adjusted for age, sex, education level, race, Townsend deprivation Index, smoking, drinking, and BMI. Model 2 is adjusted for the covariates in model 1 + NCDs including hypertension, CHD, COPD, diabetes, cataract, asthma, stroke, migraine. Model 3 is adjusted for the covariates in model 2 +depression and physical activity.


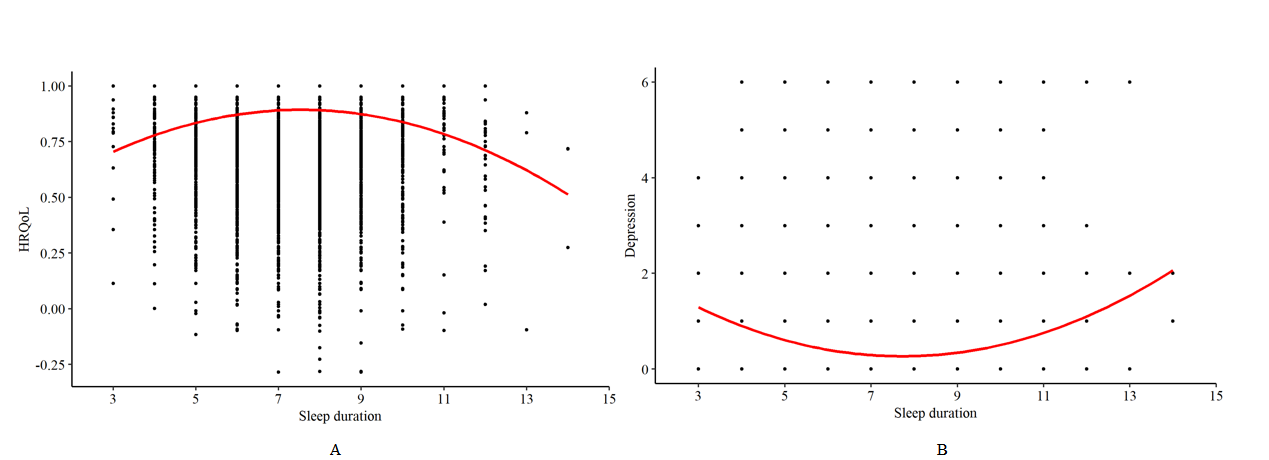


Figure s5. Quadratic effect of sleep duration on HRQoL (A) and sleep duration on depression (B).

Note, HRQoL, health-related quality of life.
